# Adaptation and evaluation of a food preference assessment tool for older adults in geriatric care: the DYSPHAGING Preferences study protocol

**DOI:** 10.1136/bmjopen-2026-120395

**Published:** 2026-09-25

**Authors:** Nicolas Massé-Deragon, Stéphanie Tripoz-dit-Masson, Coline Gréhalle, Fabien Subtil, Héloïse Rouze, Cécile Grilot, Salima Merazga, Karine Goldet, Olivier Durlach, Sophie Schir, Marine Haution-Bitker, Marion Merdinian, Claire Falandry

**Affiliations:** 1 Institut du Vieillissement, Hospices Civils de Lyon, Lyon, France; 2 CRC Vieillissement Cerveau Fragilité, Hospices Civils de Lyon, Lyon, France; 3 Speech therapist team - Centre Hospitalier de Pierre Garraud, Hospices Civils de Lyon, Lyon, France; 4 Service de Biostatistique, Hospices Civils de Lyon, Lyon, Auvergne-Rhône-Alpes, France; 5 CNRS, UMR5558, Laboratoire de Biométrie et Biologie Evolutive, Universite Claude Bernard Lyon 1, Villeurbanne, France; 6 Pôle de Santé Publique, Hospices Civils de Lyon, Lyon, France; 7 Laboratoire Reshape, Université Claude Bernard Lyon 1, Lyon, France; 8 Dietitian team - Centre Hospitalier Pierre Garraud, Hospices Civils de Lyon, Lyon, France; 9 Direction à la Recherche en Santé, Hospices Civils de Lyon, Lyon, France; 10 Centre de Recherche Clinique Vieillissement Cerveau Fragilité - Hôpital des Charpennes, Hospices Civils de Lyon, Villeurbanne, France; 11 Geriatric department - Centre Hospitalier Pierre Garraud, Hospices Civils de Lyon, Lyon, France; 12 Geriatric department - Centre Hospitalier Lyon Sud, Hospices Civils de Lyon, Lyon, France; 13 CarMeN Laboratory, Inserm U1060, INRA U1397, Université Claude Bernard Lyon 1, INSA Lyon, Charles Mérieux Medical School, University of Lyon, Oullins, France; 14 Geriatric department - Centre Hospitalier de la Croix-Rousse, Hospices Civils de Lyon, Lyon, France

**Keywords:** GERIATRIC MEDICINE, Organisation of health services, Protocols & guidelines, Frail Elderly, NUTRITION & DIETETICS

## Abstract

**Introduction:**

Undernutrition is a major concern in older adults due to its multifactorial origins and its impact on morbidity and mortality. Oropharyngeal dysphagia is common in frail geriatric patients and contributes to undernutrition by prompting individuals to progressively withdraw foods from their diet, leading to dietary imbalance and potential complications. Although texture adaptation and nutritional strategies are widely implemented in clinical practice, little is known about the actual food preferences of hospitalised older adults with dysphagia. Existing tools to assess food preferences are scarce and poorly adapted to the heterogeneity and sensory impairments of this population. The DYSPHAGING Preferences study aims to develop a food-preference assessment tool specifically tailored to hospitalised older adults, assess its test–retest reliability and describe food preferences measured using this adapted instrument.

**Methods and analysis:**

The DYSPHAGING Preferences study is designed as a prospective, multicentre, non-comparative protocol consisting of three steps. Step 1 will adapt a forced-choice questionnaire—initially developed for paediatric populations—to adults aged≥70 years hospitalised in geriatric wards, by assessing visual and lexical recognition of pre-selected food items. Step 2 will evaluate the test–retest reliability and practical feasibility of administering the preference questionnaire in a new cohort of older adults. Step 3 will be a cross-sectional study describing food preferences and comparing Preference Indexes between participants screening positive and screening negative for dysphagia using the Eating Assessment Tool-10. The questionnaire will include six categories of food (acidic, textured, sweet, in sauce, dairy and cooked). A total of 209 participants will be included across acute geriatric, rehabilitation and long-term care units (35 participants in step 1, 35 participants in step 2 and 174 participants in step 3, with the 35 participants from step 2 included in the step 3 population). Descriptive and comparative analyses will be performed according to a predefined statistical analysis plan.

**Ethics and dissemination:**

The study protocol was approved by the Comité de Protection des Personnes (CPP) Île-de-France III on 11 September 2025 (approval number: 25.02467.000911–2025-A00795-44), in accordance with French legislation. All participants will provide informed non-opposition in accordance with national regulations. The results of the primary and secondary objectives will be disseminated through peer-reviewed publications, conference presentations and communication with clinical stakeholders.

**Trial registration number:**

NCT05734586.

**STRENGTHS AND LIMITATIONS OF THIS STUDY**

- Three-step design including questionnaire adaptation, reliability assessment and multicentre evaluation.
- Recognition of food items is verified before preference assessment to ensure valid comparisons.
- Preference indexes are calculated only from food pairs recognised by participants.
- Picture-based forced-choice administration minimises cognitive and linguistic demands.
- Exclusion of participants unable to complete the questionnaire may limit generalisability.

## Introduction

 The WHO identifies healthy ageing as a major public health priority for 2016–2030.^1^ Ageing is associated with a progressive decline in various physiological functions that can lead to a gradual risk of sarcopenia, malnutrition, dysphagia, osteoporosis and frailty.^2^ Managing malnutrition remains a major challenge in older adults due to its multifactorial origins and its impact on morbidity and mortality.

Swallowing disorders—or oropharyngeal dysphagia—are recognised contributors to malnutrition.^2^ They gradually lead patients to withdraw certain foods from their diet,^3 4^ resulting in dietary imbalances^5^ and increased cardiovascular risks.^6 7^ Two European societies (the European Society for Swallowing Disorders and the European Union Geriatric Medicine Society) have defined recommendations that include raising awareness of OD, the use of screening scores, preventive measures, diagnostic standardisation and interventions such as rehabilitative training or texture adaptation.^8^ Among the available screening tools, the Eating Assessment Tool-10 (EAT-10) is a simple, validated questionnaire widely used to identify patients at risk of OD.^9^ Recently, the DYSPHAGING Pilot Study was designed to implement such a standardised care pathway in geriatric wards, combining systematic OD screening using the EAT-10 with preventive management measures.^10^

Current guidelines for undernutrition emphasise increasing energy and protein intake,^11^ but qualitative aspects of food delivered are less often considered. Studies in geriatric populations show preferences for fruits, vegetables and fish and reduced consumption of dairy and meat products.^12–14^ In people with presbyphagia or dysphagia, preferences tend to orient toward texture-modified foods or foods finely cut into small pieces.^15^ However, hedonic aspects remain understudied and the combination of dietary restriction with the consumption of texture-modified foods can lead older patients to nutritional deficiencies.^16^

In this context, strategies are needed to better tailor food intake to the preferences of geriatric patients. In adult populations, individual differences in texture^17–20^ and taste perceptions^6 21 22^ have been demonstrated and impact food appreciation. In addition, and more specifically in an older population, polypharmacy, smell, mood or cognitive disorders and OD are common and may influence food appreciation. Existing food preference assessment tools include computer-based paradigms such as the Leeds Food Preference Questionnaire, which have been widely used to investigate food reward and hedonic responses in research settings. However, these approaches are often IT-based and poorly adapted to older patients due to cognitive, praxis or visual limitations.^23 24^ Among the existing approaches to assess food preferences, the Child Food Texture Preference Questionnaire (CFTPQ) is based on a forced-choice methodology that has demonstrated good psychometric properties in a paediatric population.^25^ This approach relies on sequential choices between alternative meal presentations previously identified by participants. Forced-choice procedures are widely used in consumer preference research^26^ and have also been applied successfully in geriatric populations to assess food preferences in patients with poor appetite, representative of hospital populations.^27^ Furthermore, several studies have reported good agreement between preferences elicited using forced-choice procedures and actual food consumption.^28 29^ These characteristics suggest that the forced-choice methodology may represent a suitable framework for assessing food preferences in older adults, provided that the foods presented are representative of older adults’ dietary habits and easily recognised by this population.

DYSPHAGING Preferences is a three-step research programme designed to develop a geriatric-adapted forced-choice food-preference tool that allows calculation of preference indexes (step 1), assess the preference indexes test-retest reliability (step 2) and explore differences in preferences according to the presence or absence of OD (step 3). Beyond the development of an adapted assessment tool, the DYSPHAGING Preferences programme aims to generate population-level data on food preferences among older adults screening positive for dysphagia. These data are intended to provide an empirical basis for the future development of food products and meal proposals specifically designed for this population.

## Methods

### Study design

This study will comprise three steps, each addressing one of the predefined objectives (figure 1). Step 1 will focus on adapting a food-preference questionnaire to a geriatric population, step 2 will assess its test–retest reliability and step 3 will evaluate associations between risk of dysphagia and food preferences in a cross-sectional multicentre design.

**Figure 1:**
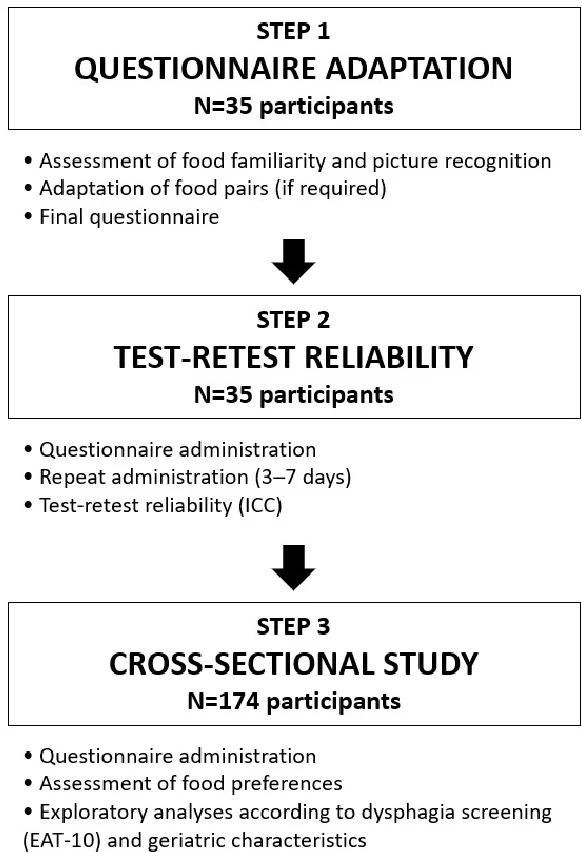
Design of the DYSPHAGING preferences protocol. ICC, intraclass correlation coefficient.

### Description of the DYSPHAGING Preferences questionnaire

The questionnaire is based on the forced-choice methodology used in the CFTPQ developed by Laureati *et al*.^25^ Following a targeted review of the literature and multidisciplinary discussions involving geriatricians, dietitians, speech-language therapists and physicians, six food preference domains were selected. These domains were chosen to reflect both food preference patterns previously described in older adults^12–14 27^ and sensory or textural characteristics considered clinically relevant in dysphagia and texture-modified diets.^6 15 16^ Selection criteria also included the expected influence of each domain on food preferences and its feasibility for assessment using picture-based forced-choice comparisons. The resulting domains were acidic, textured, sweet, in sauce, dairy and cooked.

An initial pool of 10 candidate food pairs was identified for each domain (60 pairs in total; table 1). This larger pool was intentionally designed to accommodate the adaptation step allowing food pairs that failed to meet the predefined recognition criteria to be excluded while ensuring that at least eight eligible food pairs remained available for each domain in the final questionnaire.

**Table 1:** Initial pool of candidate food pairs submitted to the adaptation step

Acidic	Textured	Sweet	In sauce	Dairy	Cooked
Red fruit salad	Beef flank steak	Grape juice	French boeuf bourguignon	Fruit yoghurt with chunks	French tomatoes ‘à la provençale’
Orange juice	Cantonese rice	Apple pie	Blanquette of veal	Plain yoghurt	Creamed spinach
Lemon meringue pie	Chocolate cake	Vanilla ice cream	Stew	Hard cheese ‘cantal’	Cooked carrots
Fish—butter and lemon sauce	Chicken breast	Chocolate cake	Spaghetti Bolognese	Cheese spread	Strawberry compote
Blackcurrant sorbet	Cooked potatoes	Fruit yoghurt	Chocolate cake-custard	Milk egg—caramel sauce	Celery puree
Lemon pie	Fruit yoghurt with chunks	Milk egg—caramel sauce	couscous	Rice pudding	Beef flank steak
Grapefruit salad	Apple pie	French croissant	Fish—sorrel sauce	Vanilla ice cream	Cooked endives
Redcurrant jelly	Hard cheese ‘cantal’	Dried fruits	Caramel pork	Glass of chocolate milk	Cheese raclette
Rhubarb pie	Vegetable soup with chunks	Rice pudding	French ‘coq au vin’	Cucumber with cream	Fruit jellies
Rhubarb compote	Hard-boiled eggs	Banana salad	Cutlet with forest sauce	Crème brûlée	Stuffed cabbage

Non-acidic	Non-textured	**Savoury**	**W**ithout sauce	Non-dairy	Raw
Peach salad	Shepherd’s pie	Tomato juice	Beef flank steak	Peach salad	Tomato salad
Grape juice	Risotto	French ‘quiche Lorraine	Lamb chop—vegetables	Apple sauce	Fresh spinach salad
Chocolate cake	Chocolate mousse	Eggplant caviar	Hamburger steak—fries	Banana salad	Shredded carrots
Fish—sorrel sauce	Chicken nuggets	Olive caviar	Plain spaghettis	Strawberry compote	Red fruit salad
Mango sorbet	Mashed potatoes	Cheese spread	Chocolate cake	Red fruit salad	Grated celery
Apple pie	Fruit yoghurt without chunks	Omelette	Plain semolina	Apple pie	Beef pie
Banana salad	Apple sauce	Baguette bread	Fish—vegetables	Mango sorbet	Endive salad
Strawberry jam	Cheese spread	Spaghetti Bolognese	Roast pork	Grape juice	Hard cheese ‘cantal’
Apricot pie	Vegetable soup	Potato chips	Chicken thigh—vegetables	Cucumber vinaigrette	Peach salad
Apple sauce	Omelette	Tomato/onion salad	Milanese cutlet	Chocolate cake	Red cabbage salad

The questionnaire will be administered in two stages (figure 2): (1) recognition, where participants will be asked whether they have previously eaten each food item (figure 2A); (2) forced choice, where they will choose between recognised items (figure 2B).

**Figure 2:**
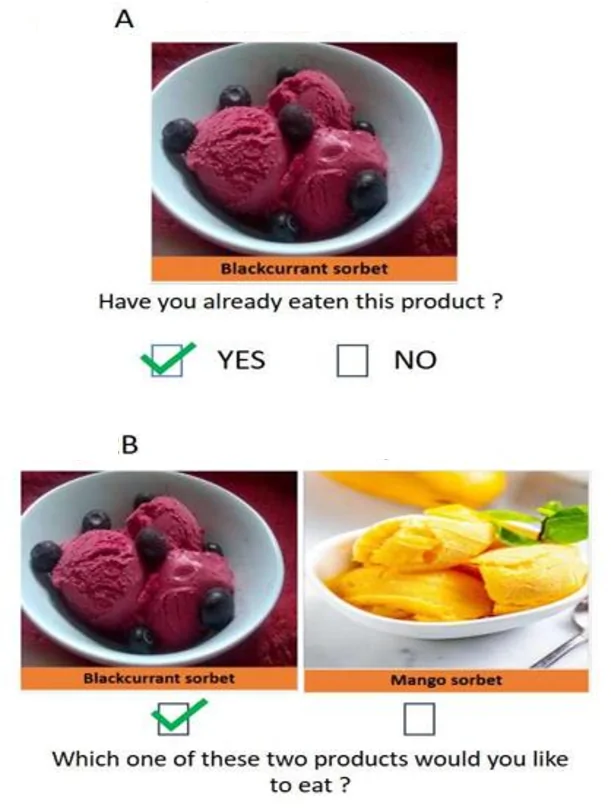
DYSPHAGING preferences questionnaire. (A) Individual recognition of each food selected by all included patients. The patient will be asked by caregivers whether he recognises the food presented. Only recognised foods will be used in subsequent analyses. (B) Forced-choice question. Pairs of selected products will be presented at the same time. The patient will be asked to choose between the two products by answering the following question: ‘Which one of these two products would you like to eat?’

The questionnaire will be administered according to a standardised procedure using identical instructions across participating centres. Investigators will receive harmonised guidance before study initiation to ensure consistent administration.

As described by Laureati *et al*,^25^ a preference index will be determined for each patient and food category as follows:


$$\begin{array}{c} \text{[(Sum of scores of valid pairs/number of valid pairs) − 1] × 100} \end{array}$$

For example, using the acidic versus non-acidic food category, when the acidic version of a food pair will be preferred, a score of 2 will be assigned. When the patient will prefer the non-acidic version, a score of 1 will be assigned for that pair. Only food pairs in which both items are familiar to the patient (i.e during the first step of the questionnaire) will be used for the index calculation.

A minimum of six valid food pairs will be required to calculate the Preference Index for a given domain. This criterion was defined to ensure that the index is based on a sufficient number of comparisons, following the same rationale as the original CFTPQ, in which participants with fewer than eight valid responses out of 17 food pairs were excluded from index calculation.^25^

### Patient population and recruitment

Participants will be recruited in acute, rehabilitation, or long-term care geriatric units (Hospices civils de Lyon, Lyon, France). Eligible patients must be aged ≥70 years, covered by health insurance, informed about the study and must not express opposition to participation. Patients unable to take food orally or unable to answer the questionnaire will be excluded. Premature withdrawal criteria will include refusal to continue, transfer before completion or death. Eligible patients will be informed about the study by an investigator or authorised team member, and non-opposition will be recorded.

### Step 1: adaptation of the questionnaire

Step 1 aims to assess item recognition and ensure suitability for older adults. A sample of 35 patients will be asked whether they have already eaten each food and whether the written description matches the image. Step 1 sample size was chosen in accordance with recommendations for questionnaire development and pretesting, which suggest that pretesting with approximately 30 participants is generally sufficient to identify problems related to item comprehension, interpretation and recognition before finalisation of the instrument.^30^ To reduce fatigue, the assessment may be divided into several sessions. Adaptation will be considered complete when at least 80% of participants recognise each food and when each category contains at least eight food pairs fulfilling the predefined recognition criteria. The 80% threshold was predefined as a conservative pragmatic criterion to ensure that retained food items and their corresponding photographs were familiar and readily identifiable to a large majority of the target population. This criterion was used to assess the suitability of the stimuli rather than their diagnostic performance. If these criteria are not met, revised items will be tested in a second cohort of 35 participants. Once the predefined adaptation criteria have been met, the questionnaire will proceed to step 2.

### Step 2: test–retest reliability

A second cohort of 35 patients will complete the questionnaire twice, 3–7 days apart. The planned sample size was considered a pragmatic compromise between the feasibility of repeated assessments in frail hospitalised older adults and the need to estimate test-retest reliability, acknowledging that domain-specific estimates may have relatively wide CIs. The retest interval was selected to minimise recall bias while limiting genuine changes in food preferences, and to remain compatible with the expected duration of hospitalisation.^31 32^ Recognition data from the first administration will define valid food pairs, and the forced-choice assessment will be repeated at retest. Preference indexes will be calculated for each domain when at least six valid food pairs are available. Questionnaire feasibility will also be assessed descriptively by recording the proportion of eligible participants refusing participation, questionnaire completion rate, administration time and the need for pauses during administration. These indicators were selected to evaluate the practical applicability of the questionnaire in hospitalised older adults. Test–retest reliability will be assessed using the Intraclass Correlation Coefficient (ICC) and its 95% CI. An ICC≥0.75, corresponding to good reliability according to Koo and Li,^33^ was predefined as the threshold to ensure that the Preference indexes provide a sufficiently reproducible basis for subsequent evaluation. Progression to step 3 will require at least four of the six domains to reach this predefined threshold. CIs will be reported alongside ICC point estimates to reflect the precision of the reliability estimates and will be considered when interpreting borderline values around the 0.75 threshold. No adaptive increase in sample size was planned for estimates with CIs spanning the 0.75 threshold. If predefined reliability criteria are not achieved, exploratory analyses may be performed to investigate response variability and to inform future refinement of the questionnaire. Collected geriatric variables and EAT-10 scores will also be used in step 3 analyses.

### Step 3: cross-sectional study

Subject to the approval of step 2, a multicentre prospective cross-sectional study will be conducted in three geriatric departments. A total of 174 participants will be included, including the 35 from step 2. Based on an expected risk of dysphagia prevalence of 31% and a moderate effect size (d=0.5) regarding the comparison between patients with and without OD, this sample will provide 80% power to detect group differences in Preference Index (two-sided α=5%). The association between dysphagia and changes in eating habits will be explored using the EAT-10,^9^ a widely used dysphagia screening questionnaire available in multiple languages, including French.^34^ Patients with an EAT-10 score≥3 will be considered as at risk of dysphagia in future analyses. Descriptive analyses will be performed to characterise food preferences in the study population. Preference Indices will be compared between participants screening positive and screening negative for dysphagia using Wilcoxon rank-sum tests. As these between-group comparisons are secondary descriptive analyses across predefined food preference domains rather than confirmatory hypothesis-testing analyses, no adjustment for multiple comparisons is planned. Findings will be interpreted cautiously, taking into account effect sizes, CIs and their clinical relevance.

### Data management and statistical analyses

Study monitoring will be performed by a Clinical Research Associate. Data inconsistencies will be communicated to investigators and corrected or recorded as missing. Adverse events will be reported according to regulatory requirements. Continuous variables will be described as mean (SD) or median (IQR), depending on their distribution, and categorical variables as counts and percentages. No imputation of missing data is planned, and analyses will be performed using available data. Feasibility outcomes (participation rate, questionnaire completion rate, administration time and need for pauses during administration) will be summarised descriptively. The intraclass correlation coefficient will be estimated with its CI, considering a single fixed-rater approach. Statistical analyses will be performed by an independent statistician using SAS and R (R Foundation for Statistical Computing, Vienna, Austria URL https://www.R-project.org). No interim analysis is planned. All statistical tests will be two-sided, with a significance level of 0.05. Effect estimates and 95% CIs will be reported whenever appropriate.

### Confidentiality

Correspondence tables linking participant identifiers to clinical data will be stored separately. Access to nominative information will be password-protected according to institutional confidentiality standards.

### Patient and public involvement

A patient partner reviewed the participant-facing study documents to improve their clarity. However, patient representatives who accurately reflect the population under study were not included in the initial development of the tools, as the target population consists of frail hospitalised patients.

### Study status

Recruitment started in October 2025. At the time of manuscript revision, step 1 (questionnaire adaptation) and step 2 (test–retest reliability assessment) have been completed, and statistical analyses of the step 2 data are ongoing. The results of the completed step are not yet available for publication. According to the study protocol, the planned study end date is November 2027.

## Discussion

Although numerous studies have explored dietary habits in hospitalised, institutionalised or appetite-impaired older adults,^12 27 35^ the DYSPHAGING Preferences project is, to our knowledge, the first to specifically assess food preferences in hospitalised older individuals with presbyphagia or swallowing disorders. Understanding these preferences is particularly relevant, as dysphagia frequently leads to dietary restrictions that may increase the risk of malnutrition, reduce meal pleasure, and compromise overall nutritional intake.

Developing a tool tailored to the older population presents several challenges. Older adults display considerable heterogeneity in their food preferences,^36^ influenced by personal history, cultural background, lifelong eating patterns^37–39^ and environmental contexts.^37^ To address this variability, we selected a forced-choice methodology, previously validated in a paediatric population,^25^ but rarely applied in geriatric settings. This approach is particularly suited to populations with potential cognitive or linguistic limitations, as it minimises verbal demands and standardises choice processes. However, adapting this method to older adults requires careful consideration, since familiarity with foods and visually recognisable items are key determinants of choice, often preceding flavour-based evaluations. Visual and sensory decline is frequent with ageing,^40 41^ and these deficits may affect food identification and food-related decision-making. For this reason, we selected visual stimuli that were highly representative and of sufficient contrast and paired them with clear written labels. The first step of the protocol was specifically designed to ensure item recognition within this population, thereby limiting selection bias and ensuring that forced-choice comparisons are meaningful. In addition, using a non-verbal recognition strategy has been shown to reduce the impact of language or literacy barriers,^42^ which are not uncommon in frail clinical populations.

Previous studies that examined food preferences in geriatric populations often evaluated only a subset of sensory or hedonic modalities, in order to minimise fatigue during assessment.^27^ While digital tools for preference testing are emerging, their use remains limited in older adults due to cognitive constraints, reduced acceptability or cost considerations.^43^ To address these limitations, we developed a low-cost, portable tool that can be administered at the bedside, and if necessary, over several short sessions to reduce participant burden.

In our pilot study, the EAT-10 was implemented in routine care to support early identification of swallowing disorders. Although a cut-off of 2 provided high sensitivity in that context,^10^ recent evidence suggests that a threshold of 3 may offer a better balance between sensitivity and specificity when compared with instrumental evaluations such as videofluoroscopic swallowing study (VFSS) or fibreoptic endoscopic evaluation of swallowing (FEES).^44^ Using this cut-off should therefore improve the characterisation of participants without dysphagia and strengthen analyses of food preferences between groups.

Given the complexity of nutritional behaviours in older adults, comorbidities and medication use will also be systematically collected. Chronic conditions such as hypertension, diabetes or dyslipidaemia often shape long-term dietary habits, while polypharmacy—common in geriatric populations—can alter taste, appetite or swallowing function.^12^ Similarly, dental status is a critical determinant of food choice and texture tolerance, independent of the presence of dysphagia,^45^ and will therefore be carefully assessed.

The DYSPHAGING Preferences study began in October 2025 and, according to the study protocol, is planned to be completed by November 2027. The questionnaire is intended to be administered as part of routine nutritional assessment by healthcare professionals involved in nutritional care. Rather than providing an individual dietary prescription, it aims to characterise food preference patterns among older adults, particularly those screening positive for dysphagia.

Ultimately, this project aims to provide a practical and patient-centred tool to better characterise food preferences in older adults with swallowing disorders. By generating robust data on food preferences in this population, the questionnaire could provide an empirical basis for the future development of food products and menu concepts specifically designed to meet both nutritional requirements and swallowing-related constraints. Although this study is expected to evaluate the reliability of the questionnaire in hospitalised older adults, further work will be required to establish its broader measurement properties, including its responsiveness and performance in other clinical settings and populations. Ultimately, intervention studies will be needed to determine whether integrating food preference data into the development of dedicated food products and nutritional care strategies improves meal acceptability, dietary intake and nutritional outcomes.

## Ethics and dissemination

The study is sponsored by the Hospices Civils de Lyon. The protocol was approved by the Comité de Protection des Personnes (CPP Île-de-France III) on 11 September 2025 (approval number: 25.02467.000911–2025-A00795-44), in accordance with French legislation. All participants receive oral and written information about the study and are included according to the French non-opposition procedure applicable to this category of research. Participants may withdraw at any time without affecting their medical care.

The study is conducted in accordance with the Declaration of Helsinki, Good Clinical Practice guidelines and the General Data Protection Regulation. Any substantial protocol amendments requiring ethical approval will be submitted to the relevant regulatory authorities before implementation.

The results of the primary and secondary objectives will be disseminated through peer-reviewed publications, presentations at national and international scientific meetings and communication with healthcare professionals involved in geriatric nutritional care.
