# Supplementary material for: Adaptation and evaluation of a food preference assessment tool for older adults in geriatric care: the DYSPHAGING Preferences study protocol
Source: BMJ Open. 2026 Sep 25;16(9):e120395. doi: 10.1136/bmjopen-2026-120395 (PMC13630011; doi:10.1136/bmjopen-2026-120395)
Supplement: Supplementary file 1 [file bmjopen-2026-120395.reviewer_comments.pdf]

PEER REVIEW HISTORY

BMJ Open publishes all reviews undertaken for accepted manuscripts. Reviewers are asked to complete a checklist review form and are provided with free text boxes to elaborate on their assessment. These free text comments are reproduced below.

ARTICLE DETAILS

Title (Provisional)

Adaptation and evaluation of a food preference assessment tool for older adults in geriatric care: the DYSPHAGING Preferences study protocol

Authors

Massé-Deragon, Nicolas; Tripoz-dit-Masson, Stéphanie; Gréhalle, Coline; SUBTIL, Fabien; Rouze, Héloïse; Grilot, Cécile; Merazga, Salima; Goldet, Karine; Durlach, Olivier; Schir, Sophie; Haution-Bitker, Marine; Merdinian, Marion; Falandry, Claire

---

VERSION 1 - REVIEW

**Reviewer**	**1**
**Name**	**Teasdale, Scott**
**Affiliation**	**University of New South Wales, Discipline of Psychiatry & Mental Health**
**Date**	**21-Jun-2026**
**COI**	**None**

This three-phase protocol aims to aims to develop, assess the reliability of, and describe food preferences measured by the adapted instrument. The planned project is interesting and likely clinically relevant–with better positioning within the manuscript. I have noted a number of comments below for review by the authors.

Abstract

1. Consider more explicitly stating that the study aims to develop, assess the reliability of, and describe food preferences measured by the adapted instrument.

2. The protocol primarily evaluates instrument adaptation, item recognition, and test-retest reliability, with only limited assessment of broader validity. I would remove the use of the word 'validation'.

3. Consider referring to participants as 'screening positive for' or 'at risk for' dysphagia given you are using the EAT-10 cut-off.

4. I would include the approving body and approval number for ethics rather than 'appropriate French regulatory authorities.'

Introduction.

5. A clearer positioning of this within existing food preference tools (their strengths and limitations) would be useful.

6. Please justify the adaption of the paediatric tool.

7. It would be useful to the more about the clinical application–who is expected to administer the tool, when and where it would be used within routine care, how the resulting preference indices should be interpreted, and what actions should follow based on the findings.

Methods

8. Please provide greater detail regarding the literature review, multidisciplinary discussions, criteria used to select domains, and whether patients contributed to the selection process.

9. The manuscript states that each category contains eight food pairs; however, Table 1 appears to contain approximately ten food pairs per category. Please clarify the final number of food pairs per domain and total comparisons completed by participants.

10. WIll there be a standardized administration protocol and training across sites?

11. Is there a minimum number of pairs required before generating the index?

12. Please justify the choice of a 3–7 day retest interval and discuss whether clinical changes occurring during this period could influence food preferences and reliability estimates.

13. Several methodological decisions in Phases 1 and 2 are presented without citation or justification, including sample sizes, the ≥80% food recognition threshold, ICC >0.75, and the requirement that at least four categories meet this threshold before progression to Phase 3. Please provide supporting rationale or references.

14. As stated in an earlier comment, consider referring to participants as 'screening positive for' or 'at risk for' dysphagia given you are using the EAT-10 cut-off.

15. The selected domains combine sensory characteristics, texture-related characteristics, and food preparation/food group characteristics. Please justify not applying multiple testing correction, given likely correlations.

16. The description of planned analyses is brief, please add greater detail regarding planned analyses, handling of missing data, assessment of statistical assumptions, and management of incomplete questionnaire responses.

17. Please clarify whether patients contributed to anything beyond review of participant-facing materials. If not, it would be useful to understand why.

18. Recruitment commenced in October 2025 and was ongoing at the time of submission. It would helpful to know the status of the study, particularly if any element has now been completed.

Discussion.

19. The discussion would benefit from more clearly acknowledging any additional work needed to establish broader validity and clinical utility, including how questionnaire results may ultimately inform patient care or nutritional outcomes.

Table 1.

20. Figure 1 appears to have grammar-check markings, please remove.

VERSION 1 - AUTHOR RESPONSE

Abstract

1. Consider more explicitly stating that the study aims to develop, assess the reliability of, and describe food preferences measured by the adapted instrument.

We thank the Reviewer for this helpful suggestion. We agree that the objectives of the study were not sufficiently explicit in the original Abstract. The sentence has been rewritten to clearly state the three sequential objectives of the study: questionnaire development and adaptation, assessment of test–retest reliability, and description of food preferences measured using the adapted instrument.

Location in revised manuscript: Abstract, Lines 45-48

2. The protocol primarily evaluates instrument adaptation, item recognition, and test-retest reliability, with only limited assessment of broader validity. I would remove the use of the word 'validation'.

We thank the Reviewer for this important comment. We agree that the term *validation* could overstate the scope of the present protocol. We have therefore amended the manuscript to reserve this term for previously validated instruments cited in the literature and replaced it, where appropriate, with more accurate terminology such as *adaptation*, *item recognition*, *eligibility according to predefined recognition criteria*, and *test–retest reliability* when referring to the DYSPHAGING Preferences questionnaire.

Location in revised manuscript: throughout the manuscript.

3. Consider referring to participants as 'screening positive for' or 'at risk for' dysphagia given you are using the EAT-10 cut-off.

We thank the Reviewer for this important remark. We fully agree that the EAT-10 is a screening instrument rather than a diagnostic tool. Accordingly, the terminology has been revised throughout the manuscript to consistently refer to participants as *screening positive for dysphagia* or *screening negative for dysphagia*, where classification is based on the EAT-10

Location in revised manuscript: throughout the manuscript.

4. I would include the approving body and approval number for ethics rather than 'appropriate French regulatory authorities.'

We thank the Reviewer for this suggestion. The Abstract and the *Ethics and Dissemination* section have been amended to explicitly report the name of the approving ethics committee together with the approval number, in accordance with BMJ Open reporting recommendations.

Location in revised manuscript: Abstract, *Ethics and Dissemination* section (Lines 64-65).

Introduction.

5. A clearer positioning of this within existing food preference tools (their strengths and limitations) would be useful.

We thank the Reviewer for this helpful suggestion. We agree that the positioning of our questionnaire within the existing literature has been strengthened.

We have amended the Introduction to provide a more balanced overview of existing food preference assessment approaches. Specifically, we now describe the strengths of the forced-choice methodology, including its established psychometric properties, previous use in consumer research and older adults, and its association with actual food consumption. We also clarify the limitations of currently available tools for frail hospitalized older adults and explain why adaptation of this methodology was required for patients with swallowing disorders. These additions better position the DYSPHAGING Preferences questionnaire within the existing literature and highlight the specific methodological gap addressed by the present study.

Location in revised manuscript: lines 116-133

6. Please justify the adaption of the paediatric tool.

We thank the Reviewer for this helpful comment. We agree that the rationale for using the Child Food Texture Preference Questionnaire (CFTPQ) as the methodological basis for the present study required further clarification. The Introduction has therefore been revised to

better explain that the CFTPQ was selected because of its forced-choice methodology, which offers several methodological advantages over traditional preference assessment tools. We also clarify that this approach was considered relevant for older adults provided that the foods presented are representative of their dietary habits and easily recognised by this population. Finally, we explicitly introduce the DYSPHAGING Preferences programme and its objective of developing a food preference questionnaire specifically adapted for hospitalized older adults.

Location in revised manuscript: Introduction lines 121-133

7. It would be useful to the more about the clinical application–who is expected to administer the tool, when and where it would be used within routine care, how the resulting preference indices should be interpreted, and what actions should follow based on the findings.

We thank the Reviewer for this important comment and agree that the intended application of the questionnaire was insufficiently described in the original manuscript.

The primary purpose of the instrument is to characterise food preferences among older adults screening positive for dysphagia at the population level. The resulting data are intended to provide an empirical basis for the future development of food products, recipes or meal options specifically designed for this population, rather than to generate an individual dietary prescription for each participant.

We have amended the Introduction and Discussion to clarify this intended application, including how the preference indices will be interpreted and how the findings may inform subsequent food development work.

Location in revised manuscript :lines 137-142 and 341-356

Methods

8. Please provide greater detail regarding the literature review, multidisciplinary discussions, criteria used to select domains, and whether patients contributed to the selection process.

Response of the author:

We thank the Reviewer for this valuable comment. We agree that the process used to develop the questionnaire and select the food preference domains required further clarification.

We have expanded the Methods section to better describe the development process. We now specify that the selection of the six food preference domains resulted from a review of the

literature combined with multidisciplinary discussions involving geriatricians, dietitians, speech-language therapists and physicians. Candidate domains were selected to reflect both food preference patterns previously reported in older adults and sensory or textural characteristics considered clinically relevant in dysphagia and texture-modified diets. We also clarify that domain selection was guided by their clinical relevance, their potential influence on food preferences and their feasibility for assessment using picture-based forced-choice comparisons.

Finally, we have clarified the extent of patient involvement. A patient partner reviewed the participant-facing study documents to improve their clarity and comprehensibility. However, patients were not involved in the selection of the food preference domains or in the development of the questionnaire, as a patient partner would not adequately represent the characteristics and clinical context of the frail hospitalized older adults targeted by this study. Consequently, these stages relied on the available literature and multidisciplinary clinical expertise.

Location in revised manuscript: lines 154-163 and 281-285

9. The manuscript states that each category contains eight food pairs; however, Table 1 appears to contain approximately ten food pairs per category. Please clarify the final number of food pairs per domain and total comparisons completed by participants.

We thank the Reviewer for identifying this lack of clarity. The initial version of the questionnaire intentionally includes ten candidate food pairs for each domain. This larger pool was designed to accommodate the adaptation phase, during which food pairs failing to meet the predefined recognition criteria may be discarded. The objective is to retain a minimum of eight eligible food pairs per domain in the final version of the questionnaire. We have amended the Methods section to clarify this distinction between the initial candidate pool and the final questionnaire.

Location in revised manuscript: lines 164-168

10. WIll there be a standardized administration protocol and training across sites?

We thank the Reviewer for this important comment. We agree that standardisation of questionnaire administration is essential to ensure consistency across study sites.

We have clarified in the Methods section that the questionnaire will be administered according to a standardised procedure using identical instructions across participating centres. Investigators involved in the study will receive harmonised guidance before participant inclusion to ensure consistent administration of the questionnaire.

Location in revised manuscript: lines 175-177

11. Is there a minimum number of pairs required before generating the index?

We thank the Reviewer for this important methodological comment. We agree that the criteria for calculating the Preference Index were not sufficiently detailed in the original manuscript.

We have now clarified that a Preference Index will only be calculated when at least six valid food pairs are available within a given domain. This threshold was selected to ensure that the index is based on a sufficient number of comparisons while limiting data loss due to unrecognised food pairs. This approach follows the same methodological rationale as the original Child Food Texture Preference Questionnaire (CFTPQ),

Location in revised manuscript: lines 186-190

12. Please justify the choice of a 3–7 day retest interval and discuss whether clinical changes occurring during this period could influence food preferences and reliability estimates.

We thank the Reviewer for this important methodological comment. We agree that the rationale for the selected test–retest interval required further clarification.

We have expanded the Methods section to explain that the 3–7 day interval was selected as a pragmatic compromise between two methodological considerations. First, the interval should be sufficiently short to minimise genuine changes in food preferences and clinical status, while reducing the likelihood that participants remember their previous responses. Second, it needed to remain compatible with the expected duration of hospitalisation in this frail older population, in whom prolonged intervals would substantially increase the risk of discharge or clinical instability.

We also acknowledge that clinical changes may occur during hospitalisation and could influence food preferences independently of measurement reliability. Although such changes cannot be completely excluded, the relatively short retest interval was chosen to minimise this risk while maintaining the feasibility of repeated assessments in routine geriatric care

Location in revised manuscript: lines 221-226

13. Several methodological decisions in Phases 1 and 2 are presented without citation or justification, including sample sizes, the ≥80% food recognition threshold, ICC >0.75, and the requirement that at least four categories meet this threshold before progression to Phase 3. Please provide supporting rationale or references.

We thank the Reviewer for this very important methodological comment. We agree that the rationale underlying the sample sizes and decision thresholds was insufficiently described in the original manuscript. We have therefore expanded the Methods section and added methodological references where applicable.

The sample of 35 participants planned for the test–retest phase was selected to provide a feasible yet sufficiently informative reliability assessment in this frail hospitalized population. This choice was informed by methodological work showing that the required sample size for ICC-based reliability studies depends on the expected ICC, the number of repeated measurements, and the desired precision or statistical power.

Location in revised manuscript: lines 205-209

The ≥80% recognition threshold was predefined as a conservative, pragmatic criterion to ensure that the retained photographs and food labels were familiar and readily identifiable to a large majority of the target population. Because this phase evaluates the suitability and recognition of the stimuli rather than a diagnostic test, this threshold was not derived from a formal diagnostic accuracy criterion.

Location in revised manuscript: lines 212-216

The criterion of ICC >0.75 was selected because ICC values between 0.75 and 0.90 are commonly interpreted as indicating good reliability.

Location in revised manuscript: lines 233-235

Finally, the requirement that at least four of the six domains meet the predefined reliability criterion was an a priori pragmatic progression rule. It was intended to ensure that a majority of the food preference domains demonstrated good test–retest reliability before proceeding to the descriptive phase, while allowing individual domains with lower reliability to be reconsidered or interpreted cautiously. We have now explicitly identified this as a study-specific decision rule rather than a universally established psychometric threshold.

Location in revised manuscript: lines 235-238

14. As stated in an earlier comment, consider referring to participants as 'screening positive for' or 'at risk for' dysphagia given you are using the EAT-10 cut-off.

We thank the Reviewer for this comment. We have amended the terminology throughout the manuscript to consistently refer to participants as *screening positive for dysphagia* (or *screening negative for dysphagia*, where appropriate) when classification is based on the EAT-10, in accordance with the role of the EAT-10 as a screening instrument rather than a diagnostic tool.

Location in revised manuscript: throughout the manuscript.

15. The selected domains combine sensory characteristics, texture-related characteristics, and food preparation/food group characteristics. Please justify not applying multiple testing correction, given likely correlations.

We thank the Reviewer for this important methodological comment. We agree that the rationale for not planning a formal adjustment for multiple comparisons should be clarified.

The primary objective of Phase 3 is to describe food preference patterns in hospitalized older adults. Comparisons between participants screening positive and screening negative for dysphagia constitute secondary analyses intended to explore potential differences across the predefined food preference domains, rather than to formally test a single confirmatory hypothesis. For this reason, no adjustment for multiple comparisons was planned. We have clarified this rationale in the Statistical Analysis section and now explicitly state that these secondary analyses will be interpreted cautiously, considering effect sizes, confidence intervals and their clinical relevance rather than statistical significance alone.

Location in revised manuscript: lines 255-259

16. The description of planned analyses is brief, please add greater detail regarding planned analyses, handling of missing data, assessment of statistical assumptions, and management of incomplete questionnaire responses.

We thank the Reviewer for this helpful comment. We agree that the Statistical Analysis section required further clarification. We have therefore amended this section to provide additional details regarding the planned descriptive and comparative analyses, the calculation of the Preference Indices, the management of missing data, the software used, and the interpretation of reliability analyses. We have also clarified the predefined decision rules applied throughout the study, as detailed in our responses to Comments 11–15.

Location in revised manuscript: lines 261-274

17. Please clarify whether patients contributed to anything beyond review of participant-facing materials. If not, it would be useful to understand why.

We thank the Reviewer for this comment. As detailed in our response to Comment 8, we have clarified the extent of patient involvement in the amended manuscript. A patient partner contributed by reviewing the participant-facing study documents to improve their clarity and comprehensibility. No further patient involvement was undertaken in the selection of the food preference domains or the development of the questionnaire, as a patient partner was not considered sufficiently representative of the frail hospitalized older adults targeted by this study. This clarification has been added to the Patient and Public Involvement section

Location in revised manuscript: lines 281-285

18. Recruitment commenced in October 2025 and was ongoing at the time of submission. It would helpful to know the status of the study, particularly if any element has now been completed.

We thank the Reviewer for this helpful suggestion. We agree that the study status should be updated.

We have amended the Study Status section to reflect the current progress of the project. At the time of manuscript revision, Step 1 (questionnaire adaptation) and Step 2 (test–retest reliability assessment) had been completed, and statistical analyses of the Step 2 data were ongoing. In addition, throughout the revised manuscript, the terminology has been harmonised by replacing the term "phase" with "step" to better reflect the sequential structure of the study and avoid confusion with clinical trial terminology. As this manuscript describes the study protocol, the study results are not presented.

Location in revised manuscript: lines 288-291

Discussion.

19. The discussion would benefit from more clearly acknowledging any additional work needed to establish broader validity and clinical utility, including how questionnaire results may ultimately inform patient care or nutritional outcomes.

We thank the Reviewer for this valuable suggestion. We agree that the Discussion should better distinguish between the objectives of the present protocol and the future work required before routine clinical implementation.

We have therefore expanded the Discussion to clarify that, although this study aims to develop and evaluate the reliability of a food preference questionnaire adapted to hospitalized older adults, additional studies will be required to establish its broader measurement properties and clinical utility. We also emphasise that the questionnaire is intended to provide population-level food preference data that may inform the future development of dedicated food products and meal proposals. Finally, we acknowledge that intervention studies will be necessary to determine whether integrating these findings into nutritional care improves meal acceptability, food intake and nutritional outcomes.

Location in revised manuscript: lines 333-356

20. Figure 1 appears to have grammar-check markings, please remove.

Response of the author:

Thank you for this suggestion. The study flowchart has been amended and simplified to provide a clearer overview of the three study phases and their respective objectives.

VERSION 2 - REVIEW

**Reviewer**	**1**
**Name**	**Teasdale, Scott**
**Affiliation**	**University of New South Wales, Discipline of Psychiatry & Mental Health**
**Date**	**19-Aug-2026**
**COI**	

Thank you for the revised version of the manuscript. The protocol is much improved.

My main follow-up point to consider is that 35 participants for test-retest reliability was chosen with both feasibility and precision in mind, however I feel that sample size may be good for exploratory analyses but may produce imprecise estimates for this type of study. As progression depends on at least four of six domain-specific point estimates needing to reach threshold, is there potential to do further work on borderline estimates (with CIs that span the threshold), i.e., could it prompt further recruitment?

Also, the statement in the abstract 'A total of 209 participants will be included across acute geriatric, rehabilitation, and long-term care units (35 in step 1, 35 in step 2, 139 in step 3).' is a little misleading given that the 35 in step 2 are included in step 3, with step 3 therefore totalling 174 participants. Could you recast the abstract to detail this more clearly.

I would also do a final grammar and citation check (e.g., include the reference citation number when writing Laureati et al – Line 183).

VERSION 2 - AUTHOR RESPONSE

TO REVIEWER

We thank Dr Teasdale for his careful reassessment of our manuscript and for his constructive comments. We appreciate his acknowledgement that the revised protocol has been improved.
Abstract

1. My main follow-up point to consider is that 35 participants for test-retest reliability was chosen with both feasibility and precision in mind, however I feel that sample size may be good for exploratory analyses but may produce imprecise estimates for this type of study. As progression depends on at least four of six domain-specific point estimates needing to reach threshold, is there potential to do further work on borderline estimates (with CIs that span the threshold), i.e., could it prompt further recruitment?

Thank you for raising this important point. We agree that, with a sample of 35 participants, confidence intervals around domain-specific ICC estimates may be relatively wide and that borderline point estimates with confidence intervals spanning the predefined threshold should therefore be interpreted cautiously, whether the point estimate lies slightly below or above 0.75. The ICC ≥0.75 threshold was predefined as the criterion for good reliability, according to Koo and Li, and progression to Step 3 requires this threshold to be reached in at least four of the six domains. This relatively demanding progression criterion was chosen to ensure that the Preference Indexes used in the subsequent multicentre evaluation would provide a sufficiently reproducible basis for further investigation.

We agree that additional recruitment could improve the precision of borderline ICC estimates. However, an adaptive increase in sample size based on the observed ICC estimates or their confidence intervals was not prespecified in the study protocol. We therefore chose to retain the predefined progression rule rather than introduce additional recruitment according to the observed reliability estimates. Confidence intervals will nevertheless be reported alongside ICC point estimates and considered when interpreting the precision and uncertainty of borderline estimates. If the predefined reliability criteria are not achieved, the protocol instead provides for exploratory analyses of response variability to identify potential sources of instability and inform future refinement of the questionnaire.

To clarify this point, the Step 2 section has been revised as follows:

« A second cohort of 35 patients will complete the questionnaire twice, 3–7 days apart. The planned sample size was considered a pragmatic compromise between the feasibility of repeated assessments in frail hospitalized older adults and the need to estimate test–retest reliability, acknowledging that domain-specific estimates may have relatively wide confidence intervals. »

And:

« Test–retest reliability will be assessed using the Intraclass Correlation Coefficient (ICC) and its 95% confidence interval. An ICC ≥0.75, corresponding to good reliability according to Koo and Li [33], was predefined as the threshold to ensure that the Preference Indexes provide a sufficiently reproducible basis for subsequent evaluation. Progression to Step 3 will require at least four of the six domains to reach this predefined threshold. Confidence intervals will be reported alongside ICC point estimates to reflect the precision of the reliability estimates and will be considered when interpreting borderline values around the 0.75 threshold. No adaptive increase in sample size was planned for estimates with confidence intervals spanning the 0.75 threshold. If the predefined reliability criteria are not achieved, exploratory analyses may be performed to investigate response variability and to inform future refinement of the questionnaire. »

2. Also, the statement in the abstract "A total of 209 participants will be included across acute geriatric, rehabilitation, and long-term care units (35 in step 1, 35 in step 2, 139 in step 3)." is a little misleading given that the 35 in step 2 are included in step 3, with step 3 therefore totalling 174 participants. Could you recast the abstract to detail this more clearly.

Thank you for pointing out this ambiguity. We agree that the previous wording could incorrectly suggest that Step 3 includes only 139 participants, whereas the 35 participants enrolled in Step 2 are also included in the Step 3 population. The abstract has therefore been revised to make the overlap between Steps 2 and 3 explicit.

The revised text reads: « A total of 209 participants will be included across acute geriatric, rehabilitation, and long-term care units (35 participants in Step 1, 35 participants in Step 2, and 174 participants in Step 3, with the 35 participants from Step 2 included in the Step 3 population). »

3. I would also do a final grammar and citation check (e.g., include the reference citation number when writing Laureati et al – Line 183).

A final grammar and citation check has been performed throughout the manuscript, and several minor grammatical and stylistic corrections have been made. Citations have also been checked throughout the text.
